# Older people's views on what they need to successfully adjust to life with a hearing aid

**DOI:** 10.1111/hsc.12016

**Published:** 2013-05

**Authors:** Timothy B Kelly, Debbie Tolson, Tracy Day, Gillian McColgan, Thilo Kroll, William Maclaren

**Affiliations:** 1 School of Education, Social Work and Community Education, University of DundeeDundee, UK; 2 School of Health, Glasgow Caledonian UniversityGlasgow, UK; 3 Audiology Department, National Health Service GrampianAberdeen, UK; 4 School of Applied Social Science, University of StirlingStirling, UK; 5 School of Nursing and Midwifery, University of DundeeDundee, UK; 6 School of Engineering and Computing, Glasgow Caledonian UniversityGlasgow, UK

**Keywords:** audiological rehabilitation, hearing aid adjustment, older people, service-user views

## Abstract

This article reports a study exploring what older people believe would enable them to adjust to and gain maximum benefit from wearing a hearing aid. A mixed methods approach was employed during 2006 involving interviews with key stakeholders, a survey across three Scottish health board areas and focus groups. Nine key stakeholders from six national and local organisations were interviewed about the needs of older people being fitted with hearing aids. In total, 240 older people belonging to three different types of hearing impaired older people were surveyed: long-term users of hearing aids, new hearing aid users, and those on a waiting list from urban and rural areas (response rate = 24%). A series of eight follow-up focus groups with 31 audiology patients was held. Health professionals appeared to neglect appropriate provision of information and overly rely on technological interventions. Of 154 older people already fitted with hearing aids, only 52% of hearing aid users reported receiving enough practical help post fitting and only 41% reported receiving enough support. Approximately 40% reported not feeling confident in the use of their aids or their controls. Older people wanted more information than they received both before and after hearing aid fitting. Information provision and attention to the psychosocial aspects of care are key to enabling older people to adjust and optimise hearing aid benefit.

## What is known about this topic

- Audiological rehabilitation is incomplete if it only includes hearing aid fitting.
- Evidence concerning efficacy of educational or counselling interventions is mixed.
- Significant problems exist in regard to availability, uptake and adherence to audiological rehabilitation programmes.

## What this paper adds

- Older people want and require information and support before and after they are fitted for a hearing aid.
- A range of audiological rehabilitation interventions is required to meet the needs of older people, which includes group-based emotional support and counselling.

## Introduction

The ability to communicate is an important component of healthy ageing and well-being (Aguayo & Coady 2001, Hallberg *et al.* 2008). Effective communication is pivotal to caring relationships, shared decision-making and personal autonomy (Worrall & Hickson 2003), and as such is essential for dignified and respectful care. Age-related hearing impairment is one of the most common impediments to communication affecting people as they age (Gussekloo *et al.* 2003). The high prevalence of hearing impairment and its impact on people’s lives are compelling reasons to intervene and provide cost effective auditory rehabilitation. Despite advances in instrumentation, understanding of the listening environment and rehabilitation science, hearing aid rejection rates among older people remain high (Knudsen *et al.* 2010).

There is consensus that providing technology alone does not meet the needs of older persons (Abrahamson 2000, Bizner 2002, Gianopoulous *et al.* 2002, Gatehouse 2003, Hickson & Worrall 2003, Hawkins 2005, Jennings 2009, Garnefski & Kraaij 2012). Other supports are required; however, the evidence base for counselling and educationally based aural rehabilitation programmes is relatively underdeveloped. Studies have focused on pre-fitting individual counselling/education interventions (Norman *et al.* 1994, Cunningham 1996, DiSarno 1997, Gussekloo *et al.* 2003, Kramer *et al.* 2005), post-fitting individual counselling/education interventions (Andersson *et al.* 1995, Benyon *et al.* 1997, Taylor & Jurma 1999, Sweetow & Sabes 2006), pre- and post-fitting individual counselling/education interventions (Brooks 1979, 1981, Alberti *et al.* 1984, Jennings 2009). Group-based approaches include post-fitting educational classes (Norman *et al.* 1995, Worrall *et al.* 1998, Northern & Beyer 1999, Abrams *et al.* 2002, Delb *et al.* 2002), auditory training (Preminger & Ziegler 2008), and a combination of one post-fitting individual counselling/education session plus a group-based educational programme (Abrams *et al.* 1992). Other literature describes and evaluates educationally based communication courses for older persons with a range of communication impairments (Jordan *et al.* 1993, Hogan 2001, Hickson & Worrall 2003, Hickson *et al.* 2006, 2007). Results of primary studies have been mixed and contradictory due to various methodological reasons, including small sample sizes, nonsensitive outcome measures, and vague intervention focus. In addition, age-related issues are often not considered in audiological rehabilitation needs of older people (Rossi-Katz & Arehart 2011).

The unclear evidence is highlighted by two systematic reviews of aural rehabilitation programmes. One focused on individual auditory training (Sweetow & Palmer 2005) the other on group aural rehabilitation programmes (Hawkins 2005). Another critical review examined the three types of rehabilitation available to older people today: hearing aids alone, hearing assistance technology and communication programmes (Laplante-Le’vesque *et al.* 2010). Hawkins (2005) concluded that group-based aural rehabilitation programmes did have short-term improvements in perception of hearing handicap, better use of communication strategies and use of hearing aids. Long-term benefits were less clear. Sweetow & Palmer (2005) concluded that there was little evidence to support the effectiveness of individual auditory training. However the studies they reviewed also had numerous limitations and internal validity threats. Laplante-Le’vesque *et al.* (2010) found evidence to support the efficacy of hearing aid provision, hearing assistance technology and communication programmes. They also highlighted that all three rehabilitation approaches have significant problems regarding availability, uptake and adherence. They suggested that the range of rehabilitation interventions needs to improve. This is echoed by Knudsen *et al.* (2010) systematic review of the impact of a patient’s journey of hearing loss and rehabilitation. Given the inconsistent findings, problems with uptake and adherence, and lack of service user involvement in developing audiological rehabilitation programmes, the primary aim of this study is to explore older adults’ perceptions of and experiences with new hearing aid use and to identify what they believed would enable them to successfully adjust to wearing a hearing aid.

## Methods

A mixed methods four-phase sequential research design was employed in 2006. Phase 1 involved semi-structured key informant interviews with professionals providing services to older people. In Phase 2, a survey of older people either on the waiting list for a hearing aid or already fitted with a hearing aid was conducted. In Phase three, we held focus groups with older audiology out-patients, and in Phase 4 a confirmatory round of focus groups was conducted. The study received ethical approval from the University and National Health Service (NHS) Research Ethics Committees and all data stored in accordance with Data Protection Act 1998. All participants at each phase of the research gave their informed consent to participate.

### Procedures for key stakeholder interviews

Semi-structured interviews were conducted with representatives from six different organisations serving older hearing disabled people. The purposive sample was selected on the basis of location of organisation (urban, remote and rural areas) and sector (NHS, local authority social work, voluntary organisations, local and national charities). All people approached for interview agreed to participate. Interviews addressed stakeholders’ perceptions of the strengths and weaknesses of services currently offered older people, what rehabilitation services older people needed to adjust to life with a hearing aid as well as the benefits and possible outcomes of effective rehabilitation. Interviews were audio-recorded and field notes were taken. Transcripts and field notes were analysed thematically. The findings from the interviews informed the development of the survey instruments.

### Survey procedures

#### Sample

A random sample was selected from patient databases of audiology departments in three health boards serving urban, remote and rural areas of Scotland. Three different groups of older patients were sampled. This included long-term users of hearing aids (fitted with hearing aids for more than 6 months); first time hearing aid users (fitted with hearing aid for less than 6 months); and older people on a waiting list for hearing aid fitting. Randomisation was achieved through systematic sampling with a random start. Using G*Power 3.1 (Faul *et al.* 2007), a total sample size of 540 was calculated to detect a moderate effect size of *f* = 0.2 in one-way 3-group analysis of variance (ANOVA) at 5% significance and 99% power. In retrospect, 99% power was too high and resulted in a much larger sample size than necessary; a sample size of 246 would have been sufficient to detect *f* = 0.2 at 80% power and 5% significance. Inclusion criteria included being at least 60 years of age, having any type of hearing loss, having no cognitive impairment, not having a terminal or life threatening illness, and speaking English. A total of 1000 postal questionnaires were distributed. A reminder letter and duplicate questionnaires were sent after 1 month to non-respondents.

### Questionnaire design

Slightly different questionnaires were developed for participants on a waiting list and those already fitted. Those already fitted with hearing aids were able to retrospectively report on what they found helpful. Those on the waiting list reported on their anticipatory needs.

The development of the questionnaires was informed by the literature and stakeholder interviews. Older hearing aid users provided feedback on early versions of the questionnaires and piloted the final draft of the questionnaire. The final instruments included demographic information (age and gender), items related to their hearing abilities (cause of deafness, length of time hearing impaired) hearing aid usage (hours/day worn, length of time having a hearing aid) and a 5-point Likert question regarding satisfaction with audiology services. Dichotomous yes/no items were developed to capture which types of information or supports aided the adjustment to wearing a hearing aid, and about the timing of supports (see Table 4 for items). Those participants who had already received their hearing aids, were asked questions concerning whether they had received enough instruction, practical information and support both before and after fitting. A series of open-ended questions allowed respondents to identify other items that would promote adjustment to hearing aid use.

### Data analysis

Descriptive statistics for all demographic data were computed using SPSS 16. After checking for equality of variances, ANOVA was used to test for differences across health boards and sample groups on appropriate variables (age, length of time on waiting list, length of time with hearing disability, hours per day wearing hearing aid). A chi-square test was used to test different response rates across groups to the yes/no questions regarding supports and information needs. The text-based qualitative data were exported into N-Vivo (QSR International Doncaster, Victoria, Australia) for analysis. Responses were coded into themes independently by two researchers and later compared for consistency before agreeing on final coding.

### Procedures for focus groups post-questionnaires

The first round of focus groups consisted of eight groups. These initial focus groups were completed to deepen understanding of the issues identified from the survey and to further identify content for the development of a rehabilitation programme for older people. Survey respondents interested in participating in a focus group were invited to return an expression of interest form separately from their completed questionnaire. Two focus groups were scheduled in each health board area and an additional two were scheduled in the remote/rural health board. All participants who indicated willingness were invited to attend the focus groups. The semi-structured focus group guide included questions regarding participants’ own hearing loss journey, helpful supports and adjustments to life with a hearing aid, and additional supports needed. In addition, results of the survey were presented and participants discussed and explored the meaning of the results. Following the analysis of the first round of focus groups, a second set of focus groups was used to confirm the findings and further explore a proposed group-based approach to audiological rehabilitation.

Responses to questions were generated and captured on a flipchart. Sessions were also audio-recorded and transcribed for content analysis using N-Vivo. The written transcripts were compared with the recordings and the flipcharts to promote accuracy and analysed independently by two researchers. Krippendorf’s (2004) approach to content analysis was utilised. We began with a pre-existing framework for investigating the transcripts. The framework included pre- and post-fitting needs (informational, support, and practical help), issues around families and family involvement, hearing problems in general, thoughts concerning a group service and issues relating to ageing. In addition, the researchers coded text outwith these areas in an effort to move beyond our own frame of reference. Transcripts were coded separately by two researchers and coding was then compared and agreed.

## Results

### Phase 1: key stakeholder interviews

Nine people were interviewed across six organisations working with and on behalf of deaf and hard of hearing communities. The six women and three men included an NHS audiologist, two social workers and an allied health professional working in a specialist local authority social work deaf service, two human service workers from voluntary organisations serving deaf and hard of hearing people, two people from deaf and hard of hearing advocacy organisations, and an executive from a large organisation. Table 1 displays the key areas that they believed should be addressed in a rehabilitation programme.

**Table 1:** Key informants' views on required rehabilitation programme content

Theme	Examples
How to receive and use services	The journey of accessing services, getting hearing aid repaired or adjusted, best practices
Communication	Communication tips and strategies, tactical hearing, lip reading, finger spelling
Psychological Issues	Building confidence, assertiveness training, self-esteem, stigma, coming to terms with hearing loss, realistic expectations, relaxation
Mechanics of hearing aid use	Inserting aid, maintenance and care of aid, how to wear aid
Advice on dealing with practical problems	Shopping, travelling, personal safety, tips for dealing with simple problems, equipment available
Education	Causes of hearing loss, environmental aids and assistive listening devices, resources available, family education, benefits of hearing aid use, lip reading
Support	Peer support, social activities

### Survey results

#### Participants

A total of 240 completed questionnaires were returned, representing a 24% response rate. As can be seen in Table 2, the mean age of the respondents was 75.3. Although the information sheet indicated that the study was for those over 60, eight people in their 50s completed the survey. Given the low response rate the eight respondents who were approaching 60 were included in the statistical analyses. This is not unreasonable given the association of age-related hearing problems as a manifestation of physiological ageing and a likely predictor of other geriatric syndromes (Gates & Mills 2005, Lopez-Torres *et al.* 2009). The mean length of time participants reported being hard of hearing was approximately 14 years, though the time ranged from 1 to 76 years. Participants included people with very long-term hearing loss as well as those with recent hearing loss due to injury or illness. Approximately 15% indicated they had age-related hearing loss, while 14% indicated that an illness caused their hearing loss. Work-related hearing loss accounted for 12%. Nearly 45% did not know the cause of their deafness, and the remaining participants indicated a combination of causes. Long-term users made up nearly 40% of the sample (*n* = 95) and new users 18% (*n* = 43). An additional 7% of the sample were hearing aid users, although they did not specify the length of time they had been using a hearing aid (*n* = 16). There were no statistically significant differences between the three groups in terms of age [*F*(2, 221) = 1.06, *P* = 0.35, or gender χ^2^(2, *n* = 223) = 1.27, *P* = 0.53].

**Table 2:** Demographic description of survey participants

	New users	Long-term users	Wait list	Missing	Total
Number of participants	43	95	86	16	240
Mean age	74.4 (SD 9.0)	76.2 (SD 8.7)	74.6 (SD 8.2)	16	75.3 (SD 8.6)
Mean reported length of time being hard of hearing	5.7 (SD 5.6), *n* = 38	18.9 (SD 18.4), *n* = 91	11.0 (SD 15.3), *n* = 71	40	13.6 (SD 16.4), *n* = 200
Gender (count, % in group)	Female 21, 48.8% Male 22, 51.2%	Female 48, 50.5% Male 47, 49.5%	Female 49, 57.0% Male 36, 41.9% Unknown, 1, 1.1%	17	Female 118, 52.9% Male 105, 47.1%
Mean length of time (years) since receiving hearing aid	0.23 years, SD 0.15, *n* = 43	11.4 years, SD 12.3, *n* = 95	NA	0	7.9 years, SD 11.4, *n* = 138
Mean hours per day using hearing aids	7.8, SD 4.9, *n* = 34	9.79, SD 6.2, *n* = 82	NA	22	9.2, SD 0.5, *n* = 116

For those on the waiting list (*n* = 86), the mean length of time being on the waiting list was 8.5 months (SD 5.5) with a range from 6 weeks to 2 years. However 31 people did not know how long they had been on the waiting list. There was a significant difference in the mean length of time on the waiting list reported by participants from the three health board areas [*F*(2, 52) = 3.39, *P* = 0.04] with the mean in months for the three health board areas being 6.5 (SD 4.6), 8.1 (SD 4.6) and 11.4 (SD 7.0) months.

#### Support needs

The 154 participants who had already been fitted with a hearing aid were asked if they had received enough instruction, practical help and support during the process of receiving a hearing aid. Fifty-one per cent (*n* = 79) of the respondents already fitted felt that they received enough instruction in the use of their hearing aid pre-fitting, increasing to 64% feeling that they received enough instruction after issue (*n* = 99). Only 52% (*n* = 80) felt they received enough practical help, and the percentage dropped to 41% (*n* = 64) when asked about getting enough support to use the hearing aid after fitting. Over one-third (36%) of hearing aid users reported not feeling confident using their hearing aids or using the controls on the aid (*n* = 56). These results were consistent across long-term and new users.

All respondents were asked whether it would be/would have been helpful to learn more about the various types of information or supports identified by the key informants. Table 3 summarises these data. There were few statistically significant differences among the three groups (see Table 4).

**Table 3:** Percentage of respondents indicating it would be helpful to learn more about key topics areas before and/or after hearing aid fitting

	Before receiving hearing aid		After receiving hearing aid	
		
Topic area	%	*N*	%	*N*
Your hearing loss	65.4	157	44.6	107
How to maintain and get the best from your hearing aid	69.6	167	63.3	152
How your ear works	52.1	125	38.3	92
Support in coming to terms with your hearing loss	47.9	115	38.8	93
General good communication skills	45.4	109	40.8	98
Assertiveness and being more confident in coping with your hearing loss	44.6	107	38.3	92
Specialised equipment that can help you cope with your hearing loss (e.g. flashing doorbells/loop systems)	42.1	101	37.1	89
Lip-reading	25.0	60	25.0	60
Finger spelling	10.4	25	11.7	28

**Table 4:** Percentage of respondents by group answering yes it would be helpful to learn more about key topics areas before and/or after hearing aid fitting*

	Long-term user		New user		Waiting list participant				
						
	%	*N*	%	*N*	%	*N*	*N*	χ^2^(2)	*P*-value
Before getting a hearing aid would it have been/would it be helpful to learn more about:									
Your hearing loss	78.7	90	73.7	38	83.3	72	200	5.11	0.08
How your ear works	56.3	87	64.9	37	67.7	65	189	2.21	0.33
How to maintain and get the best from your hearing aid	78.7	89	71.4	35	94.1	68	192	10.38	0.01
Support in coming to terms with your hearing loss	55.8	86	56.8	37	59.7	67	190	0.24	0.89
General good communication skills	57.6	85	50.0	37	57.8	64	183	0.67	0.72
Lip-reading	40.7	86	17.1	35	23.8	63	184	8.51	0.01
Finger spelling	16.5	85	11.4	35	9.5	63	183	1.64	0.44
Assertiveness and being more confident in coping with your hearing loss	52.3	86	54.3	35	57.8	64	185	0.45	0.80
Specialised equipment that can help you cope with your hearing loss (e.g. flashing doorbells/loop systems)	56.0	84	30.6	36	53.8	65	185	7.01	0.03
After getting a hearing aid would it have been/would it be helpful to learn more about:									
Your hearing loss	55.8	86	48.5	33	66.7	57	176	3.52	0.20
How your ear works	52.9	85	41.9	31	55.6	54	170	1.56	0.46
How to maintain and get the best from your hearing aid	78.7	89	77.4	31	93.1	58	178	6.11	0.05
Support in coming to terms with your hearing loss	55.4	83	41.4	29	58.9	56	168	2.47	0.29
General good communication skills	59.1	88	34.5	29	63.5	52	169	6.97	0.03
Lip-reading	43.7	87	16.7	30	24.1	54	171	10.09	0.01
Finger spelling	19.8	86	10.0	30	11.1	54	170	2.71	0.25
Assertiveness and being more confident in coping with your hearing loss	51.2	86	45.2	31	55.8	52	169	0.88	0.64
Specialised equipment that can help you cope with your hearing loss (e.g. flashing doorbells/loop systems)	57.5	87	19.4	31	51.8	56	174	13.57	0.001

* Totals do not add to 240 as many respondents only indicated ‘Yes’ on the questionnaire, rather than indicating ‘No’ on items they did not feel would be helpful.

Two questions asked about family involvement. Only 14% (*n* = 34) of all respondents had a family member or close friend with them during the process of being tested for or getting a hearing aid. Only 13% (*n* = 32) reported feeling that a family member or close friend should have been more involved. No waiting list participants had or wanted family involvement.

Forty-two per cent of respondents indicated they would be happy to learn about hearing loss and hearing aids in small groups, 49% indicated they would not and 9% did not respond. Comparing responses across the three groups suggested some variations in feelings about group support, though the variations were not statistically significant, [χ^2^(2, *n* *=* 203) = 5.48, *p* = 0.07]. Table 5 summarises these variations, and it is worth noting that over half of the long-term users would be happy to learn in a group and almost half of the respondents on a waiting list. However, fewer new users indicated that they would be happy to learn in a group. A post hoc analysis comparing only current hearing aid users was conducted and a significant difference between long-term users and new users was found, [χ^2^(1, *n* *=* 129) = 5.24, *P* = 0.02].

**Table 5:** Happy to learn in a group?

			Group			
			
			Long-term user	New user	Waiting list participant	Total
Happy to learn in a group?	Yes	Count	46	12	36	94
		% within Group	51.7%	30.0%	48.6%	46.3%
	No	Count	43	28	38	109
		% within Group	48.3%	70.0%	51.4%	53.7%
	Total	Count	89	40	74	203

### Post-questionnaire focus groups

#### Participants

The mean age of the 14 men and 17 women participating in focus groups was 74.8 (SD 7.9) and they ranged in age from 60 to 87 years. These participants were self-selecting participants, which may have biased the results of the focus groups. The groups consisted of people with and without hearing aids, and the mean length of time they had been hard of hearing was 16.7 years (SD 20.9) and the range was from 1 year to 74 years. Approximately half of the group participants had already been fitted with a hearing aid, some recently receiving their hearing aid (within weeks) and others were long-term wearers.

#### Needs prior to hearing aid fitting

The primary need prior to hearing aid fitting was information. Many participants described a lack of information about hearing aids generally and the process of receiving audiological services.

There’s lack of information about the pluses and minuses about wearing hearing aids and how they fit or how long it takes you, should you wear these things, how you should clean them, how you should look after them. There’s absolutely zilch information. Focus Group 1

When probed about the kinds of information people needed participants highlighted differences between NHS and private dispensers, between digital and analogue hearing aids, and the importance of understanding the causes of deafness and of having realistic expectations.

#### Differences between NHS/private dispensers and analogue versus digital

There was confusion concerning analogue versus digital aids. People either thought that the NHS did not provide digital hearing aids or they were disappointed when they found out that the NHS digital aids were conspicuous.

…I’d certainly like to know the difference between the digital hearing aid and the thing that people have … over their ears and attached to their glasses … I went to [Company] I was invited there and they weren’t very helpful because they were just promoting one thing and said that it would cost somewhere between £700 and £7000, I said well what do I get for that? A car to carry it in? He said no but they are sophisticated and we’ve done a lot of research on this and so on and so forth well maybe, maybe I don’t know but it would be helpful if I could get an unbiased opinion on hearing aids. Focus Group 2

Respondents spoke of how private dispensers would pressure them into buying a hearing aid and without complete information they felt unsure about their options.

You would have thought he was selling double glazing. You sit in there and he says the sad thing is both your ears are quite bad. I had already had a hearing test at [NHS Hospital], you’ve got a loss in both your ears but today if you were to buy them today we’d knock six hundred off and if you take the two of them at one thousand four hundred and it’s a bargain. I did hear that, I did hear the one thousand four hundred. Focus Group 3

Disappointment when hearing was not ‘normal’ again

Participants described having unrealistic expectations concerning the difference a hearing aid would make. Some thought that their hearing would be ‘normal’ again and were very disappointed when they received their hearing aid.

### Needs after fitting

Participants articulated numerous needs post fitting. In addition, many of the current hearing aid users continued to experience difficulties or lacked basic information about wearing, maintaining and getting the most out of the aid. The needs they identified were categorised as informational needs and support needs.

#### Informational needs post fitting

The need for information post fitting included the following: information on environmental aids, how to care for and maintain the aid, coping with new sounds, other sources of information and support, managing the controls, when to wear the hearing aid and communication tips. Those who had been fitted with hearing aids reported a lack of information to help them adjust to wearing them. Both new and experienced users reported the dearth of information.

You know I can’t help but compare this with the support from the diabetes. It’s just unbelievable that so little is given for audiology where with diabetes you are inundated with information…They look after you and it seems to me that with hearing aids you just have to look after yourself. It’s unbelievable that information is not available. Focus Group 3

A consistent need identified by focus group participants was information about environmental aids. Even experienced hearing aid users were unaware of the range of environmental aids available or how to access them. The most commonly discussed assistive devices were loop systems, telephones, doorbells, televisions, alarm clocks and safety devices (e.g. smoke detectors). Even some of the participants who knew about loop systems had never tried to use them.

The second most discussed informational need was related to hearing aid cleaning. This included knowing how to clean the aid and tubing, dealing with condensation, worries about getting the aid wet in the rain and caring for or changing batteries. Although some participants reported getting an ‘owner’s manual’ with their aid that included some of this information, most reported not remembering receiving such information. Participants described feeling overwhelmed when being fitted with a hearing aid, and getting home not remembering what the audiologist had told them.

Another important informational need was advice on when they should wear the hearing aid. Some participants were afraid that wearing it too much would ‘weaken’ their hearing. Participants asked if it was okay to wear the aid at night. Others were confused about the accommodation and adjustment period immediately after getting the hearing aid. Stories exemplifying the shock and discomfort of using a new aid in high noise situations (e.g. stadiums) were plentiful, and participants believed they were avoidable had people received appropriate information.

### Support required post fitting

Support needs included psychological, practical and problem-solving needs. Items included follow-up, help with adjustment period, managing problems with the aid, coping with cosmetic concerns, managing batteries, problems with inserting the aid, support to persevere with the aid, coming to terms with hearing loss and the need to wear a hearing aid, and finally assertiveness and confidence.

#### Help with device-related problems

Respondents described many difficulties with their aids. Problems included the sound of the wind blowing in the microphone, a piercing whistling sound when inserted, difficulty reassembling the aid after cleaning, uncomfortable ear pieces, ear infections, difficulties using assistive devices, electronic security devices interfering with the aid and difficulty changing batteries. Participants were unaware that most problems could be resolved.

#### Need for follow-up

Participants expressed the need for more follow-up services from the audiology department.

#### Cosmetic concerns

Some participants had cosmetic concerns and were very self-conscious about wearing their aids. Participants suggested support needs in the adjustment process of wearing the devices.

#### Family involvement

Most questionnaire respondents reported that family members were not involved and most did not wish for family involvement. For some focus group participants families had been the source of their referral to audiological services, putting pressure on them to see their doctor. Others reported that family members treated their hearing loss as a joke or it was the cause of family tensions. Participants identified reasons why family members should be involved and these included help with remembering and/or understanding what information was provided by the audiologist, understanding the problems that the hearing loss has caused, and needing different communication strategies (e.g. facing each other when speaking). The participants also identified barriers to family involvement. Barriers included the participants’ concerns regarding paternalistic treatment by their spouse or children. Other barriers originated from the communication partner (e.g. not seen as a serious illness, family too busy). There was a consensus that a person with hearing loss should be given the opportunity to bring a family member along, and that written information should be provided for family members.

## Discussion

The impetus for this study was to explore what older people thought would enable them to gain most benefit from hearing aids, including the possibility of group-based audiological rehabilitation. In interpreting our findings we acknowledge the relatively low response rate. The low response rate may have been exacerbated by unexpected patient record database challenges at research sites. For example, the patient database at one site was not current and many patients were listed in the wrong category, had wrong addresses, had died, or not informed they were on a waiting list. The low response rate requires that the results and subsequent discussion be read with caution. Respondents may represent those most dissatisfied with the service they received, have fewer functional limitations, and belong to a more vocal and articulate subpopulation. There are inherent limitations in using self-selection as a sampling strategy for focus groups and the likely positive predisposition towards group work. Still understanding the perspectives of those who have an expressed preference for auditory rehabilitation may point to key facilitators. The methodological limitations have to be offset against the innovative use of a sequential research design, which incorporated multiple methods. This methodological approach allowed for the identification of important aspects of audiological rehabilitation grounded in the views of older people, experts practising in the field and in professional literature. Such an approach could be beneficial in developing rehabilitation for other health conditions.

A key finding is that the informational needs of older people were not being met. Most of the older people in our study wanted more informational support than they received both before and after hearing aid fitting. These findings on unmet informational support needs are echoed in the professional literature which calls for more than technological interventions – providing information is commonly identified (e.g. Tolson 1997, Gatehouse 2003, Jennings 2009). However, the literature is not clear about the best timing of information or its format. Respondents in this study indicated that a differential approach to information giving would be preferred. Information prior to fitting should be preparatory in nature and post-fitting information of a more practical and immediate nature (e.g. how to use environmental aids, care for and maintain the aid, coping with new sounds and communication tips). The differential needs across the rehabilitation journey are consistent with findings from Knudsen *et al.* (2010) systematic review of correlates of hearing aid use and satisfaction. Interventions impact differentially across the rehabilitation journey. Further study about the best way to provide information support is required. It is of particular concern that respondents reported being taken advantage of by private companies and this highlights the dilemma for consumers in interpreting marketing materials in the absence of objectively presented information.

Respondents were unequivocal that more support through the formal network was needed (i.e. follow-up from NHS). Peer support is cited in the literature as an important component to adjusting to a hearing aid (e.g. Bizner 2002). However, only 42% of survey respondents stated that they would be happy to learn about hearing loss and using a hearing aid in a group. Focus group participants suggested that there were barriers to group participation for older people with a hearing impairment, but if a rehabilitation group would *clearly* meet a pressing concern, they would more likely attend and benefit. Further research is needed to identify effective means of providing support as part of a rehabilitation programme. Online support and information, peer mentoring, better designed information packages, and well-timed individual support and service user-led community based programmes are possible means of providing the support.

The audiological literature discusses the importance of involving communication partners in rehabilitation (e.g. Tolson 1997, Hogan 2001, Preminger & Meeks 2010). Interestingly, very few participants had or wanted family involvement. Focus group participants explained that communication partners were sometimes seen as less than helpful regarding their hearing impairment. However, they also recognised the need for information to be provided to family members to increase the likelihood of their future support.

Current best practice guidelines call for rehabilitation beyond hearing aid fitting alone. However given current resources, many audiology departments struggle to meet demand for hearing aid fitting alone – let alone comprehensive rehabilitation services. A group-based approach may be a feasible way to provide follow-up services in stretched services. A group service could be structured so that the majority of service users are provided with information and support maximising the benefits of hearing aid fitting and preventing problems. Only those needing extra support or follow-up could be referred on for more intensive follow-up services. This is consistent with the findings of Laplante-Le’vesque *et al.* (2010) critical review which found that a range of rehabilitative interventions should be available.

## Conclusion

This study endorses calls for enhanced auditory rehabilitation, and the findings are relevant to and consistent with messages from the international literature. Our work highlights that the perceived lack of pre- and post-fitting information and post-fitting support are unacceptable. Older people raised concerns about their ability to make informed choices between private and NHS providers in the absence of objective information and highlighted the distress associated with NHS waiting list uncertainty. We conclude that information provision and attention to psychosocial aspects of care are key to enabling older people to adjust and optimise hearing aid benefit, and that group rehabilitation approaches may offer an acceptable alternative for some older people. Older service users can and should be involved in shaping such services.
